# Etiology and Treatment of Puerperal Eclampsia

**Published:** 1905-03

**Authors:** Felix S. Martin

**Affiliations:** Beaumont, Texas


For Texas Medical Journal.

### Etiology and Treatment of Puerperal Eclampsia.

BY FELIX S. MARTIN, M. D., BEAUMONT, TEXAS.

The cause or causes of eclampsia has always been and is to this day, a question of the greatest speculation. In this brief paper I shall attempt to give a short summary of the etiology and treatment. That the question is one of importance, will be attested by any one who has seen cases of this frightful malady. Among the many theories advanced as to the cause may be mentioned: (1) albuminuria, with its accompanying causes; (2) structural change in the nerve centers and their envelopes; (3) cerebro-spinal congestion; (4) a neurosis caused by reflex irritation of the spinal system originating in the uterus; (5) general or cerebral anemia; (6) uremia; (7) ammonemia; (8) urinemia; (9) extractive matters in the blood, such as creatin, creatinin, etc.; (10) defective renal action, due to pressure of the gravid uterus upon the superior vena cava, upon the iliac veins and upon the kidneys—later supplanted by the theory of pressure on the ureters; (11) brain diseases; (12) soluble toxic ptomaines; (13) ergot, one-half ounce as a cause in one case; (14) uneliminated organic constituents of the urine; (15) heredity; (16) Dewees taught that they were epileptic, apoplectic or hysterical—a strong determination of the blood to the brain being a premonitory system; (17) Hodge: that it was due to congestion of the blood vessels of the brain; (18) Traube-Rosenstein taught the opposite: that it was due to cerebral anemia, caused by compression of the cerebral vessels from serous effusion which escaped from the vessel during the high arterial tension; (19) toxemia—the theory that is accepted today as the cause of eclampsia, but it is admitted that there is great room for research yet as to the cause. Among predisposing causes may be mentioned all acute and chronic kidney diseases. Retention of the urine, as in twin pregnancies, hydramnios, etc. By small pelves and by large fetus or fetal head, eclampsia occurring in 11 per cent of multiple pregnancies and in only 1 per cent of single pregnancies. Primiparis have it 3 to 1 multipara.

The exciting causes are abrupt suppression of urine, constipation, painful contractions of the uterus, rigid os, etc., extended and exhaustive expulsive efforts and excessive emotions.

As eclampsia, in regard to time of occurrence, may be ante-partum, intra-partum or post-partum, the treatment resolves itself into the treatment of these three stages. In order to prevent an attack of ante-partum eclampsia, the treatment is necessarily prophylactic, and the treatment of an actual attack is called curative treatment.

First, in order to prevent an attack of ante-partum eclampsia, one must recognize the symptoms which denote an impending attack. Of course, if albumen in large quantities is found in the urine, our suspicion should at once be aroused, but it must not be forgotten that eclampsia occurs in from 9 to 16 per cent when there is no albumen whatever in the urine. Hence, there are other symptoms which must be looked for. They are: high arterial tension with accelerated pulse, digestive disturbances, constipation, headache, and disturbances of vision, temporary loss of consciousness, anorexia, and a decrease of all excretions, both solid and liquid. All of these symptoms may be present with or without albumen in the urine. Another and more important constituent of the urine, especially as regards pre-eclampsic conditions, is urea. When urea is decidedly diminished, with or without the above symptoms, we should expect an eclamptic seizure at any time. The retention of urea in the system leads to the belief that the convulsions of eclampsia were uremic in origin, which has been shown to be erroneous. Large quantities of urea have been injected into rabbits without causing any toxic symptoms whatever; the injection of bile proved to be nine times as toxic as urea, but that the quantity of urea excreted is a good index of kidney inadequacy, goes without saying. Yet it must still be borne in mind that eclampsia has occurred in cases in which the amount of urea excreted was normal in quantity. Bouchard's experiments along this line are interesting and instructive, and they prove that other material exists in the urine equally poisonous, if not more so, than urea. He found that a certain quantity of normal urine would produce toxic symptoms when injected into rabbits of a given weight. But in renal insufficiency, in which there is retention in the blood of poisons, he found that a larger quantity of urine was required to produce toxic symptoms. Hence, the toxemic theory of eclampsia. Bouchard's further experiments showed that in renal insufficiency the poisons retained in the blood were due (1) to food-nitrogenous, such as meat, etc.; (2) bile; (3) decomposition of food in the intestines and reabsorption of its products (Edgar). No doubt fetal metabolism adds also to the intoxication of the mother, as shown by the fact that eclampsia occurs more often in twin than in single pregnancies. However, others claim that this is due to more pressure on the ureters than in single pregnancies.

Having recognized the symptoms, then the treatment is in order. All authors, I believe, are agreed that the first thing to do is to put the patient on an absolute milk diet. The liver, kidneys, skin, bowels and lungs must be improved in action, and the uterus evacuated if necessary. To act on the liver and kidneys, and, in fact, all the secretions, calomel is recommended, followed by salines. Calocynth and aloes are given in daily doses. Warm baths and pilocarpine—the latter in the pre-eclamptic state in cases without heart complications only. Some cases must be confined to bed; others can take moderate exercise. High enemata of pure, warm water or magnesia sulphates are recommended. An examination of the urine every few days to note quantity of albumen and urea. In these cases, Basham's mixture is especially good for the anemia. Diuretics are also indicated such as spirits nitre dulcæ, squills, digitalis, and calomel. After bowels are well emptied, pill of aloin, strychnine, and belladonna with cascara sagrada are useful to prevent return of constipation. Venesection has been advised, but there is much speculation as to its utility.

In the treatment of an eclamptic seizure, authors are agreed that chloroform anesthesia is the best procedure. The convulsions are to be controlled by chloroform inhalations and by chloral and bromide of potassium by the rectum, the latter in large doses; hypodermics of fluid extract of veratrum viride, 10 to 20 minims, to reduce arterial tension, and pulse should be kept as low as 60 by repeating the veratrum. Venesection is advised in plethoric and robust patients, and some advise it in all cases, claiming that it improves the pulse. From eight to sixteen ounces of blood are removed. There is a difference of opinion as to the use of morphine, but most authors advise from one-fourth to one-half grain, hypodermically, to control convulsions. Sweating must be aided by means of the hot bath and hot pack. In favorable cases the convulsions cease, and a comatose condition supervenes, to be followed by consciousness later. In unfavorable cases consciousness does not return, the patient dying in a state of coma.

In addition to the above treatment, most authors, and especially American, advise evacuation of the uterus. Various methods are adopted. Some prefer to rupture the membranes and leave it to nature, while the majority prefer to evacuate the uterus at once, either by instrumental dilatation of the cervix, and the application of forceps, or by version. Edgar advises incision of the cervix in case of rigid os. He makes four incisions—two lateral above and two lateral below.

The table of Green, of the Boston Lying-in Hospital for eight years, gives the following mortality in eclampsia: Ante-partum is the most fatal—mortality of 46 per cent; intra-partum is next —mortality rate of 25 per cent, and post-partum is last—mortality rate of 7 per cent. (Edgar.)

If you will pardon the length of this paper, I shall endeavor to report one of the several cases I have seen in Beaumont:

Mrs. R., age 17, primipara, seven months pregnant, was suddenly seized with eclampsia about the middle of January, 1903. I was called about 4 o'clock a. m., the first time I had seen the patient. I found her with a typical case of ante-partum eclampsia; face and eyelids swollen, together with considerable anasarca of the lower extremities; bowels constipated; urine scanty. She was having convulsions one after another, during one of which the tongue was bitten, and was now swollen. She was unconscious between attacks. I immediately ordered chloroform inhalations, and returned to the office for instruments and to secure consultation. Dr. Wier accompanied me, and when we arrived found that the chloroform had prevented the return of decided convulsions, although she was still having muscular twitchings. We gave one-half grain morphine hypodermically, followed a moment later by fifteen minims of fluid extract of veratrum viride hypodermically and chloroform inhalation continued. In the absence of a syringe, we did not give chloral and bromide per rectum. The hypodermics and chloroform controlled the convulsions perfectly, and we advised immediate evacuation of the uterus, which was finally consented to. Dr. Wier performed the operation, dilating the cervix by the combined instrumental and manual method; applied forceps, and delivered a dead fetus, under deep chloroform anesthesia. As there was no return of the convulsions, the chloroform was gradually withdrawn, and instructions left to immediately begin it in case symptoms of convulsions should return. The veratrum brought down the pulse splendidly, and it was not repeated. The convulsions ceased from that time on, and complete consciousness returned after remaining two days in a state of coma and semi-consciousness. An examination of the urine was made in the afternoon following operation in the morning, and found loaded with albumen—nothing else was looked for, and no microscopical examination was made. Purgatives, diuretics and warm baths were ordered, and all acted well. The urine was examined daily. The albumen gradually disappeared until the urine became perfectly normal. Patient continued to improve, appetite returned, and she was gradually able to resume solid food; milk diet finally discontinued. In about ten days she was allowed to get up and sat in a chair in the room, and in a few days went about the house apparently perfectly well, although weak. In about five days after getting up she had a severe chill; the urine became suddenly dark and bloody—so much so that malarial hematuria was suspected. There was intense pain in the bowels, and about on a level, and a little below the kidneys. Very little, if any, pain in the back. There was severe nausea and vomiting, so much so that medicines and food could scarcely be retained. There was very little fever—the temperature not going above 101° F., and at times no fever at all. A chemical examination of the urine revealed albumen in abundance, and on microscopical examinations numerous casts were found. The casts were of the hyaline and granular variety. A diagnosis of acute parenchymatous nephritis was made. As the vomiting and pain in the bowels were the most prominent symptoms, medicines for their relief were in order. She was given all forms of stomach sedatives, and the skin over the epigastrium was reddened with mustard. All failed to relieve the vomiting, and all the rest she got was from hypodermics of morphine. Just as soon as the effects of the morphine were gone the vomiting returned together with the pain in the bowels, and each recurrence seemed more severe. The urine continued of a reddish color and was loaded with albumen and casts.

Patient continued to grow weaker, and for days at a time was unable to retain nourishment by the stomach whatever. Resort was had to rectal alimentation, and was kept up, but the vomiting and pain in the bowels seemed to wear her out. At first she was able to retain a little buttermilk for a short while, but this was promptly ejected later, and as I said before, days and weeks passed without anything being retained by the stomach.

It is remarkable how long this woman lived in that condition. Finally, about a month from the initial chill, death closed the scene.

The points of interest are: That this patient had no treatment prior to the eclamptic attack; second, the promptness with which she recovered from the attack after the uterus was emptied; third, the stubbornness of the nephritis after the kidneys had become normal, and the intense pain in the bowels instead of in the lumbar region.

